# Tracheostomy Before Polatuzumab Vedotin–Containing Therapy for Airway‐Threatening Cervical Diffuse Large B‐Cell Lymphoma: A Case Report of Two Patients

**DOI:** 10.1155/crh/4447338

**Published:** 2026-09-28

**Authors:** Yoshikazu Ikoma, Nobuhiro Kanemura, Rinto Otsuka, Daisuke Okamoto, Naoki Hayase, Takuro Matsumoto, Nobuhiko Nakamura, Hiroshi Nakamura, Takenori Ogawa, Masahito Shimizu

**Affiliations:** ^1^ Department of Hematology and Infectious Disease, Gifu University Hospital, Gifu, Japan, gifu-u.ac.jp; ^2^ Center for Nutrition Support and Infection Control, Gifu University Hospital, Gifu, Japan, gifu-u.ac.jp; ^3^ Department of Otolaryngology-Head and Neck Surgery, Gifu University Graduate School of Medicine, Gifu, Japan, gifu-u.ac.jp

**Keywords:** airway obstruction, case report, diffuse large B-cell lymphoma, polatuzumab vedotin, tracheostomy

## Abstract

**Background:**

Bulky cervical diffuse large B‐cell lymphoma (DLBCL) with impending airway compromise presents an urgent management dilemma, particularly when diagnostic confirmation and timely initiation of definitive therapy must be balanced against the risk of abrupt airway deterioration. Evidence guiding airway‐first management before polatuzumab vedotin–containing frontline therapy remains limited.

**Case Presentation:**

We describe two older men with bulky cervical DLBCL who presented with progressive airway‐related symptoms and marked upper airway narrowing on endoscopic and radiologic evaluations. In both cases, semiurgent tracheostomy was prioritized over emergent radiotherapy or immediate systemic therapy, allowing pathological confirmation and treatment planning under controlled conditions. After DLBCL was pathologically confirmed, full‐dose polatuzumab vedotin, rituximab, cyclophosphamide, doxorubicin, and prednisone (Pola‐R‐CHP) were initiated 15 and 10 days after tracheostomy, respectively, and delivered without dose reduction or treatment delay. No perioperative or tracheostomy‐site complications occurred. Both patients completed six cycles of Pola‐R‐CHP followed by two cycles of rituximab. One patient achieved a partial metabolic response; after receiving salvage chemoimmunotherapy followed by CD19‐directed chimeric antigen receptor T‐cell therapy, he achieved a complete metabolic response. In the other patient, residual fluorodeoxyglucose uptake without corresponding structural lesions was managed with close surveillance after negative follow‐up evaluations, including laryngoscopy and thyroid fine‐needle aspiration.

**Conclusions:**

Although limited to two patients, these cases suggest that proactive airway stabilization can create a controlled window for diagnosis and treatment planning and may facilitate safe delivery of full‐dose Pola‐R‐CHP in selected patients with airway‐threatening cervical DLBCL.

## 1. Introduction

Diffuse large B‐cell lymphoma (DLBCL) is the most common subtype of non‐Hodgkin lymphoma and remains potentially curable with frontline chemoimmunotherapy [1]. Early progression still occurs, and bulky symptomatic disease can create urgent management dilemmas when immediate morbidity reflects local mass effect, requiring clinicians to balance diagnostic certainty, treatment timing, and procedural safety.

Lymphoma‐related airway obstruction is infrequently reported, and available evidence remains largely limited to case reports and small series. In patients with a predicted difficult airway, preservation of spontaneous ventilation and preparation for invasive airway access are important considerations [2, 3]. Published cases describe emergency tracheostomy for upper airway obstruction [4], bronchoscopic interventions for central airway obstruction [5], and venovenous extracorporeal membrane oxygenation (VV‐ECMO) as a bridge to diagnosis and chemotherapy in life‐threatening obstruction [6]. These reports illustrate the principle that immediate airway security can be decisive, but they do not define optimal sequencing for contemporary frontline regimens.

Polatuzumab vedotin combined with rituximab, cyclophosphamide, doxorubicin, and prednisone (Pola‐R‐CHP) has emerged as a frontline option after the Phase 3 POLARIX trial demonstrated improved progression‐free survival compared with R‐CHOP, with broadly similar overall safety [7]. In clinical practice, patients with airway‐threatening mechanical compromise may require sequential decisions: securing the airway, confirming the diagnosis, and initiating definitive therapy. However, reports focusing on Pola‐R‐CHP after airway stabilization in this setting appear limited, leaving uncertainty about feasibility and safety.

We report two older men with bulky cervical DLBCL who presented with symptoms of impending airway compromise and underwent semi‐urgent tracheostomy before receiving full‐dose Pola‐R‐CHP. We focus on two practical aspects of this sequence: the role of semiurgent tracheostomy in creating a controlled window for diagnostic confirmation and treatment planning, and the feasibility of subsequently delivering full‐dose Pola‐R‐CHP as frontline therapy after airway stabilization.

## 2. Case Presentation

### 2.1. Case 1

In 2023, a 63‐year‐old man with no relevant medical, family, or psychosocial history and no regular medications was referred to Gifu University Hospital with impending upper airway compromise after 2 months of progressive right parotid‐region swelling followed by hoarseness and dysphagia. On arrival, oxygen saturation was 98% on room air. He had no respiratory distress or obvious abnormal breath sounds at rest but experienced dyspnea when supine. Physical examination revealed bulky bilateral cervical masses with supraclavicular lymphadenopathy. Flexible laryngoscopy demonstrated a tumor extending from the right oropharynx to the hypopharynx and larynx, with glottic inlet compression (Figure 1A). Computed tomography (CT) showed marked pharyngeal narrowing due to mass effect, with a minimum residual airway lumen of approximately 12.6 × 4.8 mm. Because these findings raised concern for abrupt airway deterioration, we prioritized tracheostomy under local anesthesia over emergent radiotherapy or immediate systemic therapy. Tracheostomy and biopsy of the pharyngeal lesion were performed the following day (Figure 2). No perioperative complications occurred.

**FIGURE 1:**
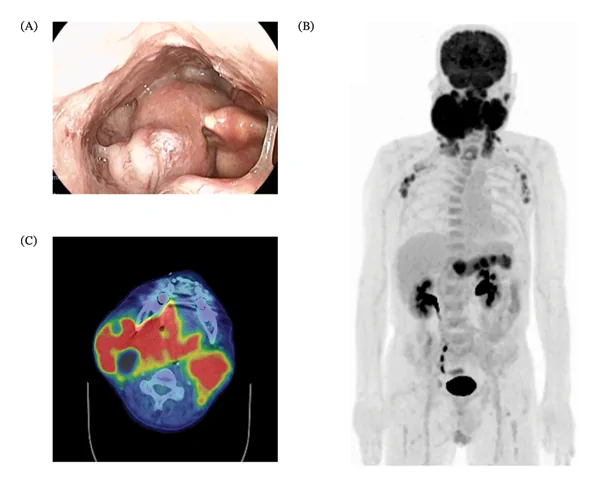
Baseline endoscopic and FDG‐PET/CT findings in Case 1. (A) Flexible laryngoscopy demonstrating compression of the glottic inlet by the tumor. (B) Maximum intensity projection FDG‐PET/CT image demonstrating extensive disease involving the palatine to lingual tonsillar region, bilateral deep cervical lymph nodes, bilateral infraclavicular/axillary lymph nodes, pancreas, and bone marrow. (C) Axial fused FDG‐PET/CT image of the neck showing the bulky cervical lesion responsible for marked upper airway compression; central photopenia within the right upper deep cervical node is suggestive of cystic change or necrosis. FDG‐PET/CT, fluorodeoxyglucose positron emission tomography/computed tomography.

**FIGURE 2:**
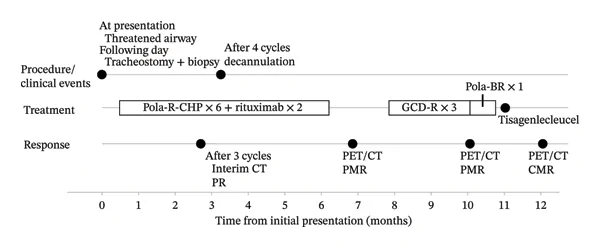
Clinical timeline of Case 1. The patient presented with a threatened airway and underwent tracheostomy and biopsy on the following day. First‐line treatment with Pola‐R‐CHP was initiated after diagnosis. Interim CT after three cycles showed partial response. Decannulation was performed after four cycles, and treatment was completed with six cycles of Pola‐R‐CHP followed by two cycles of rituximab. End‐of‐treatment PET/CT demonstrated partial metabolic response. The patient subsequently received salvage chemoimmunotherapy with GCD‐R, followed by Pola‐BR and CD19‐directed CAR‐T therapy with tisagenlecleucel, and achieved a complete metabolic response on follow‐up PET/CT. Pola‐R‐CHP, polatuzumab vedotin, rituximab, cyclophosphamide, doxorubicin, and prednisone; GCD‐R, gemcitabine, carboplatin, dexamethasone, and rituximab; Pola‐BR, polatuzumab vedotin, bendamustine, and rituximab; CAR‐T, chimeric antigen receptor T‐cell; CT, computed tomography; PET/CT, positron emission tomography/computed tomography; PR, partial response; PMR, partial metabolic response; CMR, complete metabolic response.

Histopathology confirmed DLBCL, not otherwise specified, which was classified as the germinal center B‐cell–like (GCB) subtype using the Hans algorithm [8]. Immunohistochemistry was negative for MYC, BCL2, and BCL6; FISH analyses for MYC, BCL2, and BCL6 rearrangements were not performed. Baseline Eastern Cooperative Oncology Group (ECOG) performance status was 1, with lactate dehydrogenase within the reference range and soluble interleukin‐2 receptor elevated. He had experienced > 10% unintentional weight loss, meeting a criterion for B symptoms [9]. Baseline fluorodeoxyglucose positron emission tomography/computed tomography (FDG‐PET/CT) demonstrated bulky cervical disease (maximum diameter 10 cm) with nodal involvement and extranodal disease in the pancreas and bone marrow (Figure 1B,C). The disease was staged as Lugano Stage IV [10]. The International Prognostic Index (IPI) was 3.

After airway stabilization, no bridging corticosteroids were administered before diagnostic confirmation. Pola‐R‐CHP was initiated 15 days after tracheostomy and delivered every 21 days at planned doses without reduction or delay (Figure 2). Tumor shrinkage was noted after the first cycle although dysphagia persisted. Interim CT after three cycles showed a partial response; no interim PET/CT was performed. Flexible laryngoscopy after four cycles confirmed tumor shrinkage and preserved swallowing function, and the tracheostomy tube was removed. He completed planned therapy without clinically significant infections, grade ≥ 3 toxicities [11], or tracheostomy‐site complications. End‐of‐treatment FDG‐PET/CT demonstrated residual uptake with Deauville score 4, classified as partial metabolic response according to Lugano criteria [10]. He subsequently received salvage chemoimmunotherapy followed by CD19‐directed chimeric antigen receptor T‐cell therapy with tisagenlecleucel, achieving a complete metabolic response (Figure 2).

### 2.2. Case 2

In 2025, a 74‐year‐old man had a history of extranodal mucosa‐associated lymphoid tissue lymphoma involving the stomach and palatine tonsil and had achieved a complete response after rituximab plus bendamustine. His history included remote ischemic stroke, diabetes mellitus, and dyslipidemia; medications included aspirin and vildagliptin; family and psychosocial histories were noncontributory. He noticed left cervical swelling and visited another hospital, where CT showed enlargement of the left thyroid lobe measuring 54.5 × 48.1 mm. Two days later, rapid progression of the swelling with enlargement to 62.0 × 55.1 mm prompted emergency transfer to Gifu University Hospital. At presentation, he reported hoarseness and pharyngeal discomfort; oxygen saturation was 100% on room air, and breath sounds were clear. Physical examination revealed bilateral cervical masses. Flexible laryngoscopy demonstrated narrowing at the levels of the oropharynx, hypopharynx, and larynx due to extrinsic compression by the tumor (Figure 3A,B). Contrast‐enhanced CT showed marked airway narrowing related to lingual tonsillar enlargement at the tongue base–hypopharyngeal level, with a minimum residual airway lumen of approximately 6.4 × 4.9 mm, as well as marked bilateral thyroid enlargement with tracheal compression (Figure 3C,D). Although emergent tracheostomy was not considered necessary at presentation because his respiratory status was stable, the rapid cervical enlargement and airway narrowing raised concern for further airway deterioration. We, therefore, prioritized semiurgent tracheostomy over emergent radiotherapy or immediate systemic therapy. Two days after presentation, tracheostomy was performed under general anesthesia together with thyroid biopsy (Figure 3E). No perioperative complications occurred.

**FIGURE 3:**
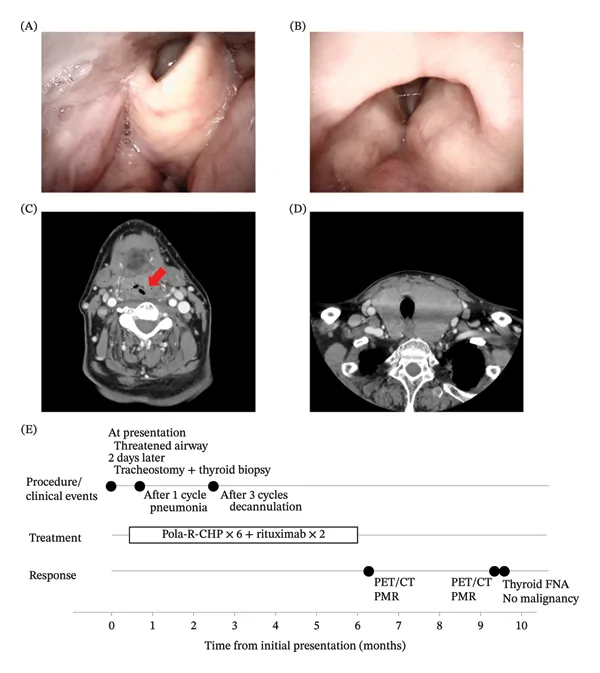
Baseline endoscopic and contrast‐enhanced CT findings and clinical timeline in Case 2. (A) Flexible laryngoscopy demonstrated narrowing at the levels of the oropharynx, hypopharynx, and larynx due to extrinsic compression by the tumor. (B) Flexible laryngoscopy showing narrowing of the laryngeal inlet caused by extrinsic compression. (C) Axial contrast‐enhanced CT image at the tongue base–hypopharyngeal level showing marked airway narrowing related to lingual tonsillar enlargement (red arrow). (D) Axial contrast‐enhanced CT image at the thyroid level showing marked enlargement of both thyroid lobes with associated tracheal compression. (E) Clinical timeline. The patient presented with a threatened airway and underwent tracheostomy under general anesthesia together with thyroid biopsy 2 days after presentation. After the first cycle of Pola‐R‐CHP, he developed Grade 2 bacterial pneumonia. Decannulation was performed after three cycles, and treatment was completed with six cycles of Pola‐R‐CHP followed by two cycles of rituximab. End‐of‐treatment PET/CT showed PMR with Deauville score 4 uptake in the thyroid and tongue base–hypopharyngeal region, without corresponding structural lesions on laryngoscopy or thyroid ultrasonography. Follow‐up PET/CT demonstrated improved but persistent tongue base–hypopharyngeal uptake and persistent thyroid uptake; repeat laryngoscopy showed no mass lesion, and thyroid FNA showed no evidence of malignancy. CT, computed tomography; Pola‐R‐CHP, polatuzumab vedotin, rituximab, cyclophosphamide, doxorubicin, and prednisone; PET/CT, positron emission tomography/computed tomography; PMR, partial metabolic response; FNA, fine‐needle aspiration.

Histopathology confirmed DLBCL, not otherwise specified, and the tumor was classified as non‐GCB by the Hans algorithm [8]. Immunohistochemistry showed MYC positivity in approximately 20% of tumor cells, negative BCL2 staining, and focal BCL6 positivity; FISH analyses for MYC, BCL2, and BCL6 rearrangements were not performed. Focal lymphoepithelial lesion‐like changes in the background follicular epithelium raised the possibility of transformation from the previous MALT lymphoma. Baseline ECOG performance status was 2, with elevated lactate dehydrogenase and soluble interleukin‐2 receptor. He had no B symptoms [9]. Although baseline FDG‐PET/CT was not performed, CT showed disease involving the tonsillar, thyroid, cervical, and upper mediastinal regions, with no lesions below the diaphragm; bone marrow biopsy showed no evidence of lymphoma involvement. The lymphoma was, therefore, classified as Lugano Stage II [10]. The IPI was 3.

Pola‐R‐CHP was initiated 10 days after tracheostomy and delivered at planned doses without reduction or delay throughout all cycles (Figure 3E). Nine days after cycle 1, he developed Grade 2 bacterial pneumonia [11], possibly related to aspiration due to bulky upper airway disease, which improved after 5 days of oral levofloxacin without delaying subsequent treatment. Tumor shrinkage was noted after the first cycle, without clear functional improvement. No interim PET/CT was performed; after three cycles, flexible laryngoscopy confirmed tumor shrinkage and preserved swallowing function, and the tracheostomy tube was removed. He subsequently completed planned therapy without tracheostomy‐site complications. Grade ≥ 3 adverse events were limited to anemia [11]. End‐of‐treatment FDG‐PET/CT demonstrated Deauville score 4 thyroid and tongue base–hypopharyngeal uptake, without corresponding lesions on laryngoscopy or thyroid ultrasonography, and we elected close surveillance. Follow‐up PET/CT showed improved but persistent tongue base–hypopharyngeal and thyroid uptake; repeat laryngoscopy showed no mass, and thyroid fine‐needle aspiration was negative (Figure 3E).

## 3. Discussion

First, semiurgent tracheostomy can create a stable window for diagnostic confirmation and treatment planning without forcing immediate cytotoxic therapy in an unsecured airway. Second, after airway stabilization, full‐dose Pola‐R‐CHP could be delivered without dose reduction or treatment delay as frontline therapy. This airway‐stabilization‐first sequence did not preclude subsequent systemic therapy. These observations remain hypothesis‐generating because they are limited to two patients.

POLARIX demonstrated improved progression‐free survival with Pola‐R‐CHP versus R‐CHOP in previously untreated DLBCL with an IPI of 2–5 [7], with exploratory analyses favoring Pola‐R‐CHP in higher‐risk patients, including those with an IPI of 3–5. Both patients had an IPI of 3, supporting Pola‐R‐CHP as an evidence‐based frontline option for their DLBCL; the regimen was administered within its reimbursed first‐line indication in Japan. This report adds practical information on the sequencing of Pola‐R‐CHP after airway stabilization in patients with airway‐threatening disease.

Most published evidence for lymphoma‐related airway obstruction consists of case reports and small series spanning heterogeneous sites and histologies. Lymphoma‐specific reports describe emergency tracheostomy for upper airway obstruction [4], bronchoscopic intervention for central airway obstruction [5], and VV‐ECMO as a bridge when ventilation becomes impossible [6]. Conversely, another lymphoma case showed rapid reversal of mechanical airway obstruction after prompt R‐CHOP despite initial intubation, suggesting that systemic therapy may be appropriate when the airway can be maintained safely [12]. More general airway‐management literature also supports awake or emergent tracheostomy and therapeutic bronchoscopy in selected patients according to anatomy, deterioration risk, and procedural feasibility [13–15]. Taken together, these reports support individualized airway management.

For bulky cervical DLBCL with threatened airway, the key initial decision is which approach most reliably prevents catastrophic obstruction while preserving systemic therapy. In both patients, progressive upper airway compromise prompted tracheostomy within 24–48 h of presentation—under local anesthesia in Case 1 and general anesthesia in Case 2—rather than emergent radiotherapy or immediate chemotherapy, consistent with difficult‐airway principles [2, 3]. Initial management, including the need for airway intervention, was guided by multidisciplinary discussion involving hematology and otolaryngology, with input from emergency medicine, radiology, and anesthesiology as appropriate. This airway‐first approach allowed diagnostic confirmation and treatment planning while reducing the risk of abrupt airway decompensation, and full‐dose Pola‐R‐CHP was feasible without dose reduction. The subsequent courses differed, and airway‐first management did not preclude salvage therapy in the first patient. Given the reported bridging strategies [4–6, 12], a practical approach includes early airway‐risk assessment and proactive stabilization when deterioration risk is high or biopsy and treatment logistics are uncertain. Multidisciplinary planning may help align airway stabilization with diagnostic confirmation and timely systemic therapy in selected patients.

This report describes only two patients and does not allow comparison of airway‐stabilization strategies or frontline regimens in airway‐threatening presentations. In addition, the cases differed in cell‐of‐origin subtype, and one had a possible background indolent component, further limiting interpretation of treatment response. Accordingly, our report should be viewed as a description of clinical decision‐making and feasibility rather than evidence of comparative efficacy or causal effect.

Overall, these two cases suggest that, in patients with bulky cervical DLBCL and impending airway compromise, semiurgent tracheostomy can create a controlled window for diagnosis and treatment planning before full‐dose Pola‐R‐CHP. Although evidence remains case based, these observations illustrate an airway‐stabilization‐first sequence for initiating polatuzumab vedotin–containing therapy in selected patients with airway‐threatening cervical DLBCL.

## Author Contributions

Yoshikazu Ikoma, Nobuhiro Kanemura, Rinto Otsuka, Daisuke Okamoto, Naoki Hayase, Takuro Matsumoto, Nobuhiko Nakamura, Hiroshi Nakamura, Takenori Ogawa, and Masahito Shimizu contributed to the conception of this report; Yoshikazu Ikoma collected the clinical data; Yoshikazu Ikoma and Nobuhiro Kanemura drafted the manuscript; Nobuhiro Kanemura, Nobuhiko Nakamura, Takenori Ogawa, and Masahito Shimizu critically revised the manuscript. Yoshikazu Ikoma had full access to all of the data in this study and takes complete responsibility for the integrity of the data and the accuracy of the data analysis.

## Funding

This work received no specific grant from any funding agency in the public, commercial, or not‐for‐profit sectors. This work was prepared as part of the authors’ employment at their respective affiliated institutions, Gifu University Hospital and Gifu University Graduate School of Medicine.

## Disclosure

All authors have read and approved the final version of the manuscript.

## Ethics Statement

This report was prepared in accordance with the CARE guidelines and conducted in accordance with the principles of the Declaration of Helsinki. Institutional review board approval was not required according to institutional policy because this report describes two deidentified patients and involved no research procedures beyond routine clinical practice.

## Consent

Written informed consent was obtained from both patients for publication of this case report and all accompanying clinical data and images.

## Conflicts of Interest

Yoshikazu Ikoma has received honoraria from AbbVie GK, Astellas Pharma Inc., Meiji Seika Pharma Co., Ltd., Nippon Shinyaku Co., Ltd., Kyowa Kirin Co., Ltd., and Chugai Pharmaceutical Co., Ltd.

Nobuhiko Nakamura has received honoraria from Chugai Pharmaceutical Co., Ltd., Kyowa Kirin Co., Ltd., AbbVie GK, Janssen Pharmaceutical K.K., Sanofi, Novartis, MSD K.K., Sumitomo Pharma Co., Ltd., Nippon Kayaku Co., Ltd., Nippon Shinyaku Co., Ltd., Meiji Seika Pharma Co., Ltd., and AstraZeneca K.K.

The remaining authors declare no conflicts of interest.

## Supporting Information

Additional supporting information can be found online in the Supporting Information section.

## Supporting information


**Supporting Information** CARE checklist.

## Data Availability

The data that support the findings of this case report are not publicly available because of patient privacy concerns. Data may be available from the corresponding author upon reasonable request and with appropriate institutional approval.
